# Identification and Bioactivities of Secondary Metabolites Derived from Endophytic Fungi Isolated from Ethnomedicinal Plants of Tujia in Hubei Province: A Review

**DOI:** 10.1007/s13659-020-00295-5

**Published:** 2021-01-20

**Authors:** Ke Ye, Hong-Lian Ai, Ji-Kai Liu

**Affiliations:** School of Pharmaceutical Science, South-Central University for Nationalities, Wuhan, 430074 Hubei China

**Keywords:** Tujia medicine, Endophytic fungi, Secondary metabolites, Bioactivities

## Abstract

Tujia is a national minority, inhabiting in the mountainous Wuling area in China. Since 1978, Tujia medicine has been studied, summarized and developed, leading to numerous achievements by Chinese researchers, such as the publishing of approximately 30 monographs of Tujia medicine. These publications are focused on summarizing and improving the theory of Tujia medicine and developing clinical therapies from this system of medicine. The shortage of natural medicinal plants used in Tujia medicine has created the need to discover new resources to replace them and protect endangered natural plant species. Endophytic fungi are one of the conservation options, are considered a source of new bioactive natural products, and are a renewable and inexhaustible source of new drugs and agrochemicals. This review summarizes 260 compounds from endophytic fungi that have been previously isolated from the medicinal plants of Tujia. These compounds include steroids, terpenoids, meroterpenoids, polyketides, alkaloids, peptides, aliphatic compounds, aromatic compounds, and heterocyclic compounds.

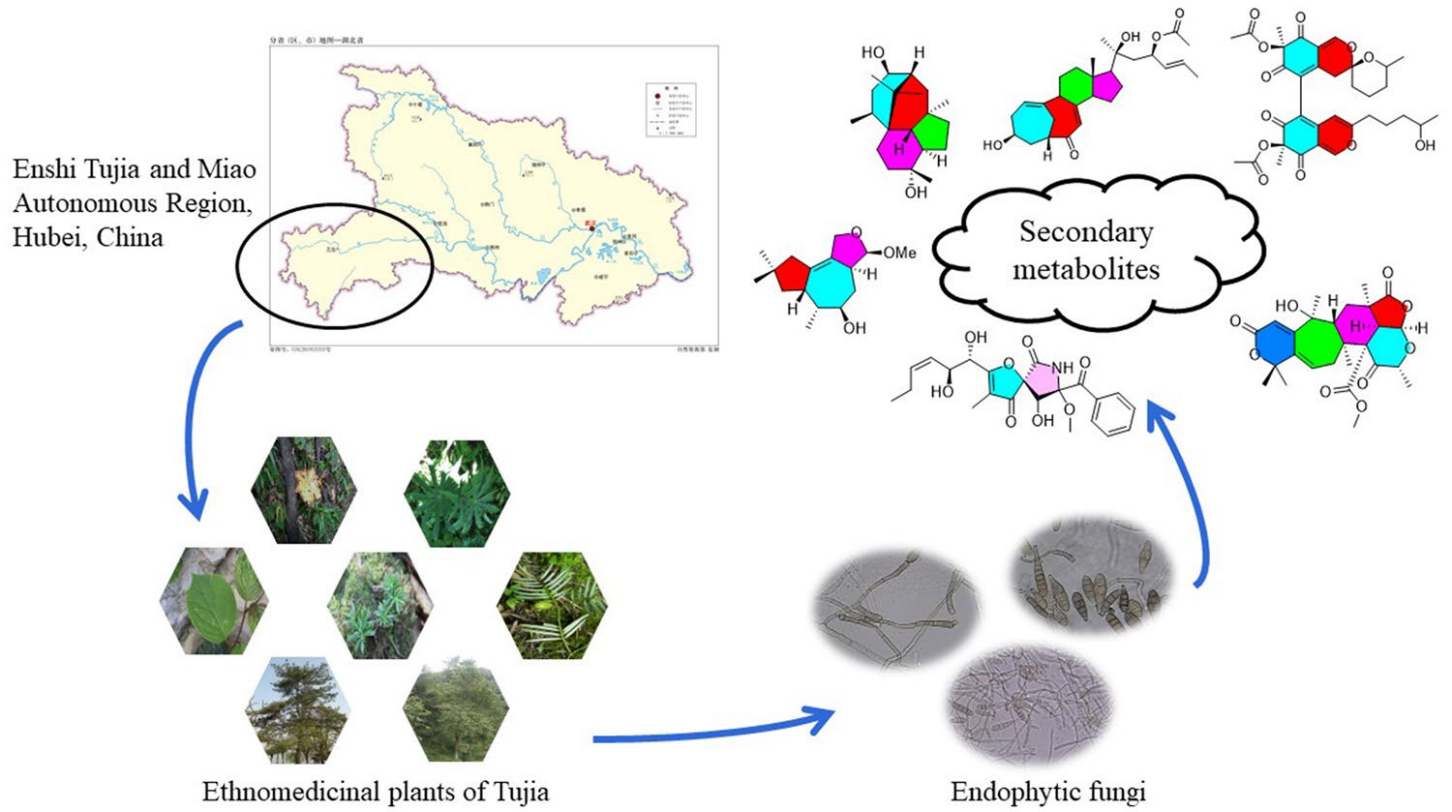

## Introduction

Endophytic fungi are microorganisms that inhabit in the inner healthy tissues of host plants. They typically do not induce any apparent symptoms of disease in the host [1]. Since anticancer agent paclitaxel (Taxol) was discovered in *Taxomyces andreanae*, an endophytic fungal strain isolated from *Taxus brevifolia* [2], interest in bioactive natural products derived from endophytic fungi has increased. During the past two decades, a considerable number of natural products with novel structures and interesting bioactivities have been reported, and endophytic fungi have been identified as to be a source of new bioactive natural products [3–5].

Interestingly, endophytic fungi can produce the same or similar bioactive metabolites as their host plants [6]. Thus, they can be used to develop a substitutable approach to producing valuable bioactive compounds to protect plant and conserve resources and the natural environment [7]. Tujia medicine is a type of Chinese medicine that has unique advantages and potential in curing different diseases, but some Tujia medicinal plants are endangered. Therefore, endophytic fungi isolated from Tujia medicinal plants of the Tujia could be a novel source of natural products, thereby protecting endangered plants.

This review summarizes metabolites, including steroids, terpenoids, meroterpenoids, polyketides, alkaloids, peptides, aliphatic compounds, aromatic compounds, heterocyclic compounds and others as well as their bioactivities of endophytic fungi isolated from the antirheumatic and anti-traumatic medicinal plant of the Tujia in Hubei province. In addition, different medicinal plant classes are described.

## *Cephalotaxus Fortunei* Hook

*Cephalotaxus fortunei* is a perennial, coniferous shrub or small tree belonging to the family Cephalotaxaceae. It is mainly distributed in the subtropical regions up to the northernmost Qinling Mountains and the Huai River in central China. *C. fortunei* contains the anticancer alkaloid harringtonine, which has made it important for medicinal use in treating leucocythemia [8].

Trichodermanin A (**1**), a novel diterpenoid with skeletal carbons arranged compactly in a fused 6/5/6/6 ring system, was isolated from the subculture of endophytic fungus *Trichoderma atroviride*, which was obtained from the bark of *C. fortune*i [9].

Two sesquiterpenes, named trichoderiols A (**2**) and B (**3**), were also isolated from cultures of the same endophytic fungus *T. atroviride* [11]. In bioactivity studies, these three compounds showed good antifungal effects against *Candida albicans*, *Cryptococcus neoformans*, and *Trichophyton rubrum* as well as some antitumor activity [10]. Furthermore, compounds **2** and **3** were evaluated for their anti-inflammatory activity against nitric oxide (NO) production and showed significant NO scavenging effects, with half-maximal inhibitory concentrations (IC_50_) values of 15.3 and 9.1 μM, respectively. The results of a 3-(4,5-dimethylthiazol-2-yl)-2,5-diphenyltetrazolium bromide (MTT) assay indicated that none of the concentrations used in the experiment were cytotoxic [11].

**Fig. 1:**
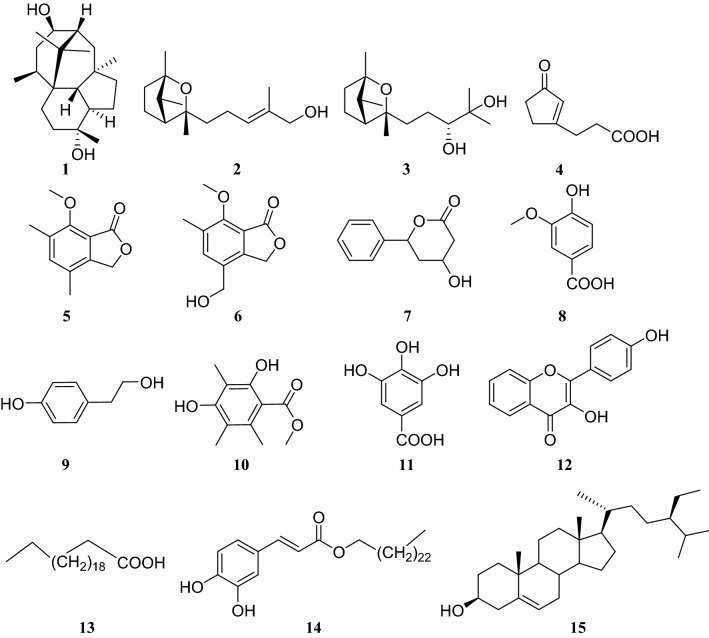
Structures of compounds **1**–**15**

**Fig. 2:**
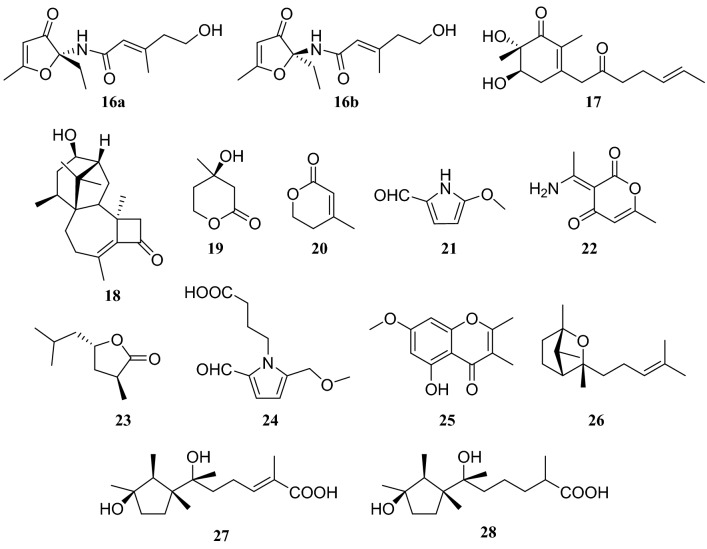
Structures of compounds **16**–**28**

In addition to terpenoids, other types of compounds have been isolated from this endophytic fungus, including 3-oxo-1-cyclopentene-1-propanoic acid (**4**), 7-methoxy-4,6-dimethyl-1(3*H*)-isobenzofuranone (**5**), 4-(hydroxymethyl)-7-methoxy-6-methyl-1(3*H*)-isobenzofuranon (**6**), tetrahydro-4-hydroxy-6-phenyl-2-pyran-2-one (**7**), vanillic acid (**8**), *P*-hydroxyethyl phenol (**9**), 2,4-dihydroxy-3,5,6-trimethylbenzoate (**10**), gallic acid (**11**), kaermferol (**12**), n-docosanoic acid (**13**), caffeic acid hexaeosanyl ester (**14**), and *β*-sitosterol (**15**) [10] (Fig. 1).

(−)-Trichodermadione (**16a**) and ( +)-trichodermadione (**16b**), two novel *N*-furanone amide enantiomers, were isolated from the solid culture of *T. atroviride* in the bark of *C. fortune*i. Moreover, trichodermadiones B (**17**) and C (**18**), a cyclohexanone sesquiterpenoid and a diterpenoid, respectively, were obtained, along with the following twelve compounds: **1**, **9**, *R*-mevalonolactone (**19**), anhydrome valonolactone (**20**), 5-methoxymethyl-1*H*-pyrrole-2-cabaaldehyde (**21**), 3-(1-aminoethylidene)-6-methyl-2*H*-pyran-2,4(3*H*)-dione (**22**), mollisilactone (**23**), 4-(2-formyl-5-(methoxymethyl)-1*H*-pyrrol-1-yl)butanoic acid (**24**), 5-hydroxy-2,3-dimethyl-7-methoxychromone (**25**), lignoren (**26**), ascotrichic acid (**27**) and catenioblin C (**28**). However, in bioactivity studies, compounds **16a**, **16b**, **17** and **18** did not show anti-inflammatory activity [12, 13] (Fig. 2).

Ma et al. [14] reported a furan derivative, 5-acetoxymethylfuran-3-carboxylic acid (**29**), which was isolated from the endophytic fungus *Aspergillus flavus* in *C. fortunei*. This compound exhibited potent antibacterial activity against *Staphylococcus Aureus*, moderate antioxidant activity, and has the potential to be an antibacterial drug.

**Fig. 3:**
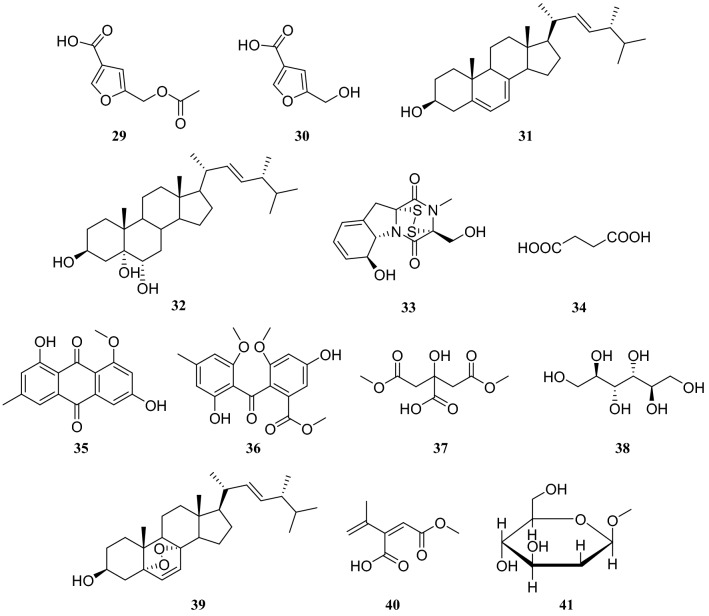
Structures of compounds **29**–**41**

Six compounds isolated from *A. flavus* in *C. fortunei* were identified as compound **15**, 5-hydroxymethyl furan-3-carboxylic acid (**30**), ergosterol (**31**), stigmasta-7,22-diene-3*β*,5*α*,6*α*-triol (**32**), gliotoxin (**33**) and succinic acid (**34**). Among these compounds, **30** and **33** showed good antibacterial activity and **30** exhibited a good inhibitory effect against *Escherichia coli*, with a minimum inhibitory concentration (MIC) of 15.6 μg/mL [15]. Bioassay-guided fractionation of the ethyl acetate (EtOAc) extract of the endophytic fungus *Aspergillus piperis* in the stem of the *C. fortunei* led to the isolation and identification of several compounds, including **31** and **33**, emodin-8-*O*-methylethe (**35**), monomehtylsulochrin (**36**), 1,5-dimethyl citrate (**37**), D-mannitol (**38**), and ergosterol peroxide (**39**), which showed antibacterial activity [16]. An endophytic fungus of the *Penicillium* sp. was collected and identified from *C. fortune*, and the following five compounds were identified as compounds **31**, **38**, and **39**, 3-isopropenyl-*Z*-butenedioic acid monomethyl ester (**40**), and methyl-*O*-*β*-D-glucopyranosid (**41**) [10] (Fig. 3).

## *Huperzia serrata* (Thunb. ex Murray) Trev

**Fig. 4:**
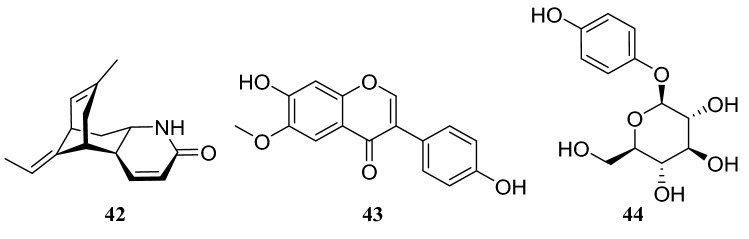
Structures of compounds **42**–**44**

*Huperzia serrata* is used in the traditional Chinese medicine preparation. Qian Ceng Ta grows at an altitude of 300–2700 m in damp forests and rock crevices in China. This plant produces the alkaloid huperzine A (**42**), which is marketed in China as a new drug for Alzheimer’s disease (AD) treatment and used in the USA as a supplement to prevent further memory degeneration [17–20]. Endophytic fungi often have the ability to produce the same or similar bioactive metabolites as their host plants. Five endophytic fungi isolated from *H. serrata* produced metabolites that were similar or identical to those of huperzine A. These endophytic fungi were characterized and identified as *Alternaria* sp., *Shiraia* sp., *Fusarium oxysporum*, and two different strains of *Colletorichum gloeosporides*. Moreover, *Alternaria* sp. produced 6-methoxy-7,4′-dihydroxyisoflavone (**43**) and arbutin (**44**) [21–25] (Fig. 4).

**Fig. 5:**
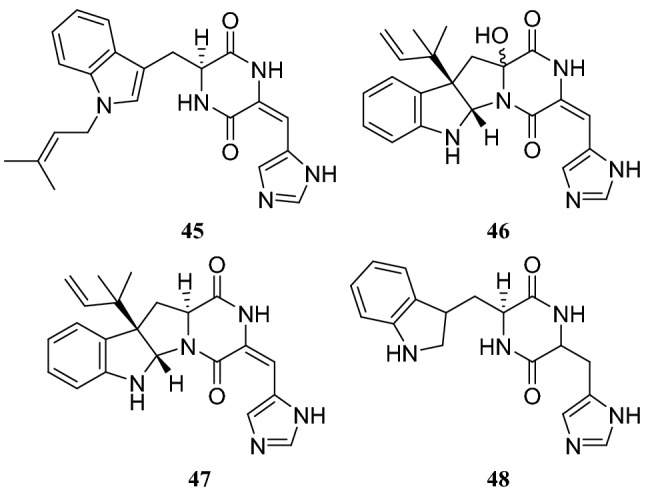
Structures of compounds **45**–**48**

A strain of the *Penicillium* sp. collected from the stem of *H. serrata* was found to produce alkaloids in preliminary experiments. Systematic experiments isolating alkaloids from the fungus led to the identification of four diketopiperazine alkaloids, tryhistatin (**45**), 16-hydroxyroquefortine C (**46**), roquefortine C (**47**), and cyclo(dehydrohistidyl-L-tryptophyl) (**48**). They are an important class of fungal metabolites and this class of alkaloids has shown great potential in the development of therapeutic drugs [26] (Fig. 5).

**Fig. 6:**
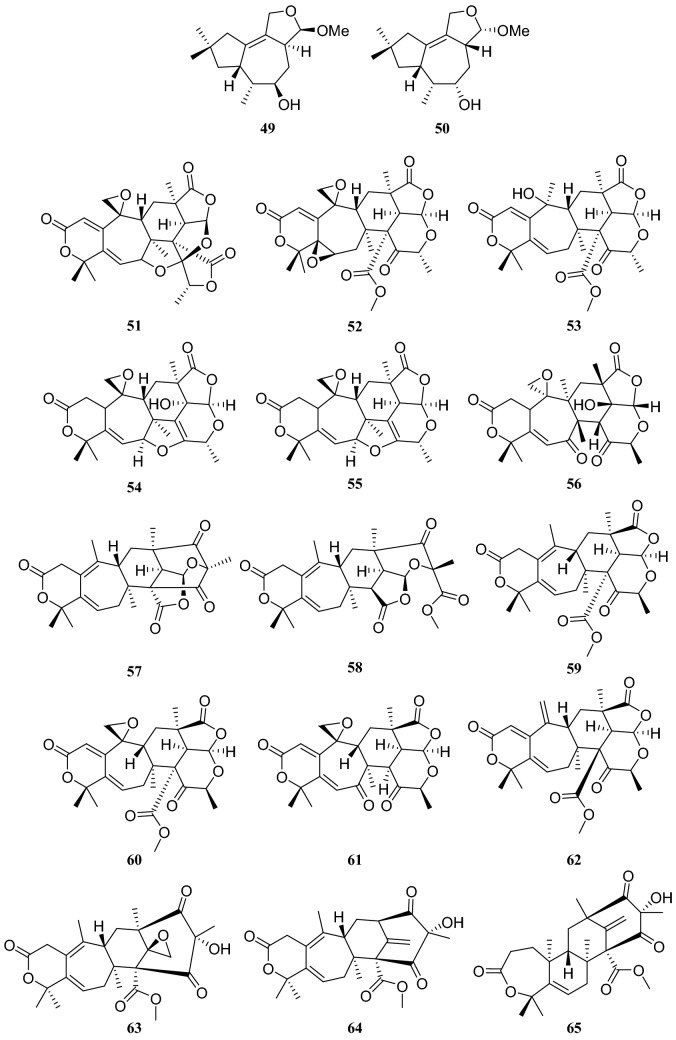
Structures of compounds **49**–**65**

Ceriponols L (**49**) and M (**50**), two tremulane sesquiterpenoids, were obtained from an endophytic fungus *Ceriporia lacerate* isolated from the stems of *H. serrata* [27]. Meroterpenoids are synthesized via a common intermediate produced by the hybridization of a polyketide intermediate and the terpenoid precursor farnesyl diphosphate. Qi et al. [28] reported fifteen 3,5-dimethylorsellinic acid derived meroterpenoids identified as chrysogenolides A–H (**51**–**58**), berkeleyacetals A–C (**59**–**61**), purpurogenolide C (**62**), 22-epoxyberkeleydione (**63**), berkelrydione (**64**), and berkeleyone B (**65**). These compounds were produced by *Penicillium chrysogenum*, an endophytic fungus collected from *H. serrata*. Among these compounds, chrysogenolides C (**53**), D (**54**), and F (**56**); berkeleyacetal C (**61**); and purpurogennolide C (**62**) showed inhibition of NO production in lipopolysaccharide (LPS)–activated RAW 264.7 macrophages with IC_50_ values in the range of 4.3–78.2 μM (Fig. 6).

**Fig. 7:**
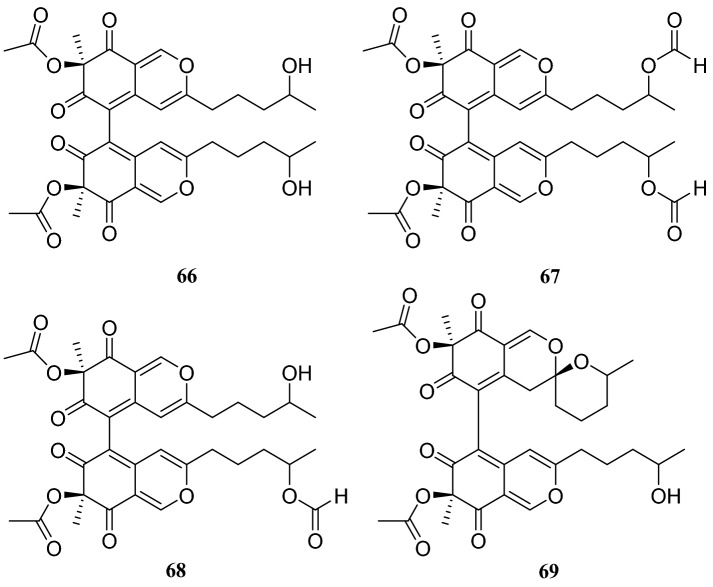
Structures of compounds **66**–**69**

Fungal polyketides are one of the largest and most structurally diverse classes of naturally occurring compounds [29]. Four new dimeric spiro-azaplilone derivatives cochliodones E–H (**66**–**69**) were obtained from an endophytic fungus of the *Chaetomium* sp., which was obtained from *H. serrata*. Furthermore, four compounds assayed and all exhibited antibacterial activity. In particular, compound **68** inhibited *E. coli* growth to levels almost the same as cefotaxime did [30]. In addition, Yu et al. [31] isolated eight compounds from an endophytic fungus of the *Chaetomium* sp., which was collected from *H. serrata*, consisting of seven polyketides and one fungal toxin. They were identified as chaetoviridine F (**70**), chaetoviridine E (**71**), (7*R*,4′*S*,5′*S*,11*S*)-chaetoviridin A (**72**), (7*R*,4′*S*,5′*R*,11*S*)-chaetoviridin A (**73**), xanthoquinodin A1 (**74**), xanthoquinodin A2 (**75**), xanthoquinodin B (**76**) and chetomin (**77**) (Fig. 7).

**Fig. 8:**
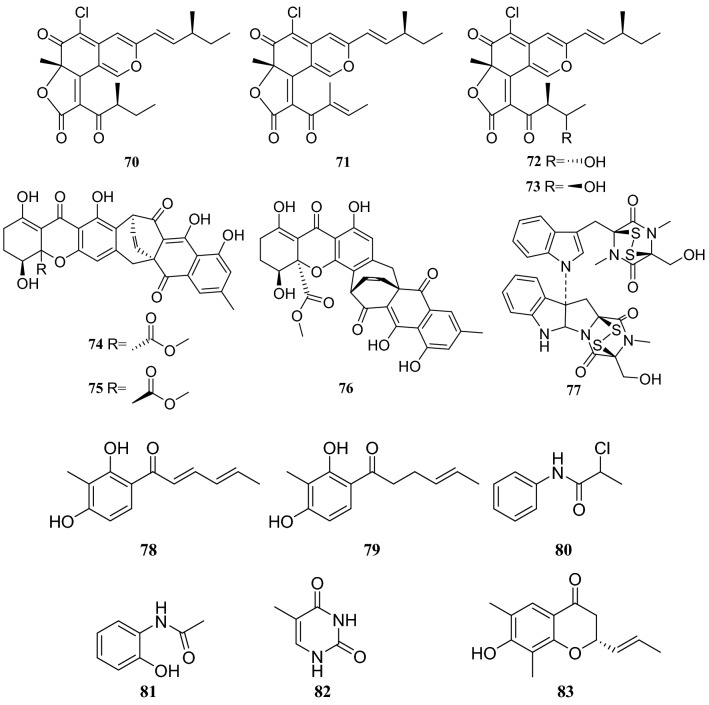
Structures of compounds **70**–**83**

Chemical investigation of the metabolites of an endophytic fungus of the *Penicillium* sp. obtained from the stems of *H. serrata*, led to the isolation of compounds **31** and **39**, sorbicillin (**78**), 2,3′-dihydrosorbicillin (**79**), 2-chloro-*N*-phenylpropranamide (**80**), *N*-(2-hydroxyphenyl)-acetamide (**81**), thymine (**82**) and a chromone derivative (2*S*)-2,3-dihydro-7-hydroxy-6,8-dimethyl-2-[(*E*)-prop-1-enyl]-chroman-4-one (**83**). Moreover, compounds **78**, **79** and **83** were subjected to an in vitro cytotoxicity assay. Compound **78** exhibited potent cytotoxicity against HeLa cells and weak activity against HepG2 cells with IC_50_ values of 1.6 and 27.2 μM, respectively. Compound **79** showed moderate activity against HeLa cells and weak activity against HepG2 cells with IC_50_ values of 7.4 and 44.4 μM [32] (Fig. 8)

**Fig. 9:**
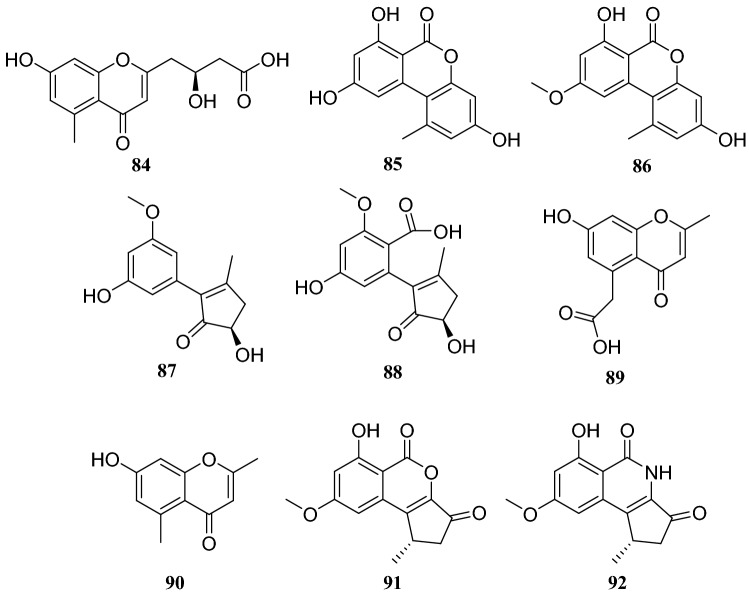
Structures of compounds **84**–**92**

Systematic investigation of metabolites from the endophytic fungus *Cercospora lagenariae* isolated from *H. serrate* led to the isolation of nine polyketides identified as cerecolagenlic acid A (**84**), alternariol (**85**), alternariol-9-methyl ether (**86**), ( +)-nigrosporal A (**87**), alternarienonic acid B (**88**), 2-methyl-5-carboxymethyl-7-hydroxylchromone (**89**), 2,5-dimethyl-7-hydroxychromone (**90)**, 1-deoxyrubrelactone (**91**) and (−)-alternarlactam (**92**). Among these compounds, **90** exhibited some inhibitory effects on NO production in LPS-activated RAW 264.7 macrophage cells, with an IC_50_ of 57.5 ± 1.2 μM [33]. Zhan et al. [34] reported the isolation and structural elucidation of six furanone derivatives, huaspenone A (**93**), huaspenone B (**94**), aspertetronin A (**95**), aspertetronin B (**96**), gregatin E (**97**) and penicilliol A (**98**). These compounds were isolated from the cultures of an endophytic fungus of the *Aspergillus* sp. obtained from the stems of *H. serrata*. Eight metabolites isolated from an endophytic fungus of the *Peyronellaea* sp. from *H. serrata* were identified as compounds **19** and **20**, glycerol 2-acetyl-3,5-dihydroxyphenylacetate (**99**), curvulinic acid (**100**), *O*-methylcurvulinic acid (**101**), methyl curvulinate (**102**), andrastin A (**103**) and fuscoatramide (**104**) [35] (Fig. 9)

Eight diphenyl ether derivatives obtained from an endophytic fungus *P. chrysogenum* isolated from *H. serrate* were identified as penicichrysogenillide A (**105**), penicichrysogenillide (**106**), talaromyone A (**107**), isopencillide (**108**), penicillide (**109**), hydroxypenicillide (**110**), purpactin A (**111**) and penicichrysogenillide (**112**). Furthermore, in additional studies, compounds **105** and **106** showed inhibitory activity against NO production in LPS-stimulated RAW264.7 macrophage cells with IC_50_ values of 76.2 and 41.2 μM, respectively [36].

**Fig. 10:**
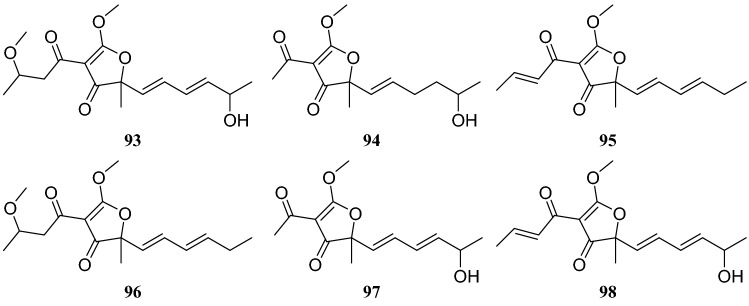
Structures of compounds **93**–**98**

Several compounds were isolated from three different endophytic fungi collected from *H. serrate*. The first strain, *Shiraia* sp. produced eburicol (**113**), 2,3-dihydroxypropyl-9*Z*,12*Z*-octadecadienoate (**114**)**,** (*R*)-2,3-hydroxypropyl stearate (**115)**, 2,3-dihydroxypropyl-hexadecanoate (**116**), linoleic acid (**117**), hypocrellins A (**118**), hypocrellins B (**119**), elsinochromes B (**120**), and elsinochromes C (**121**). In bioactivity studies, compounds **114**–**116** and **118** showed antibacterial activity. The second strain *Aspergillus fumigatus* produced compound **31**, dioctyl phthalate (**122**), 1′,9,12-linoleic acid-2′,3′-dihydroxypropyl ester (**123**), microsphaerone C (**124**), and helvolic acid (**125**). Moreover, compounds **123**–**125** exhibited inhibitory activity against acetylcholinesterase (AChE) with rates of 73.5%, 84.1% and 77.6% and IC_50_ values of 0.057, 0.038, and 0.05 mg/mL, respectively. The third strain of the *Neofusicoccum* sp. produced compounds **31**, **39**, cerebroside C (**126**), fusaproliferin (**127**), adenosine (**128**), 1-(furan-2-yl)-hyroxyethanone (**129**), versicolactone A (**130**), and versicolactone B (**131**) [37–39] (Fig. 10)

## *Pinus massoniana* Lamb

*Pinus massoniana* is widely grown in Asia and is an important source of timber and oleoresin in southern China [40]. An endophytic fungus of the *Phomopsis* sp. was isolated from *Pinus massoniana* and nine compounds were isolated, which consisting of four nonenolides, three alternariol derivatives, and two phthalide derivatives. The systematic names of the four nonenolides are (5*S*,8*S*,9*R*,10*R*,*E*)-5,8,9-trihydroxy-10-pentyl-3,4,5,8,9,10,-hexahydro-2*H*-oxecin-2-one (**132**), (5*S*,8*S*,9*R*,10*R*,*E*)-5,8,9-trihydroxy-10-nonyl-3,4,5,8,9,10,hexahydro-2*H*-oxecin-2-one (**133**), (5*S*,6*S*,9*S*,10*R*,*E*)-5,6,9-trihydroxy-10-pentyl-3,4,5,6,9,10-hexahydro-2*H*-oxecin-2-one (**134**), and (5*R*,8*S*,9*R*,10*S*,*E*)-5,8,9-trihydroxy-10-([*R*]-4-hydroxyoctyl)-3,4,5,8,9,10-hexahydro-2*H*-oxecin-2-one (**135**).

The two phthalide derivatives were 3,5-dihydroxy-7-mehtoxy-4-(methoxymethyl)-6-methyl-isobenzofuran-1(3*H*)-one (**136**) and 5-hyroxy-3,7-dimethoxy-4-(methoxymethyl)-6-methyl-isobenzo-furan-1(3*H*)-one (**137**). Three alternariol derivatives are compounds **85**, **86**, and alternariol 4,10-dimethyl ether (**138**). All compounds were evaluated for antitumor and antibacterial activities, but none of them showed obvious activity [41]. Another endophytic fungus of the *Glomerella* sp. was obtained from *P. massoniana*, and two lanostane-type triterpenoids and four steroid derivatives were isolated.

**Fig. 11:**
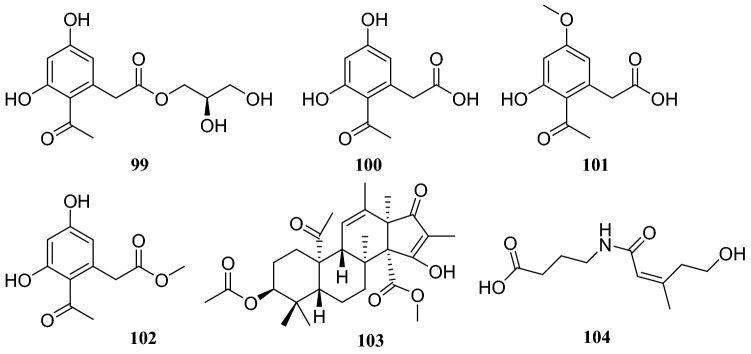
Structures of compounds **99**–**104**

**Fig. 12:**
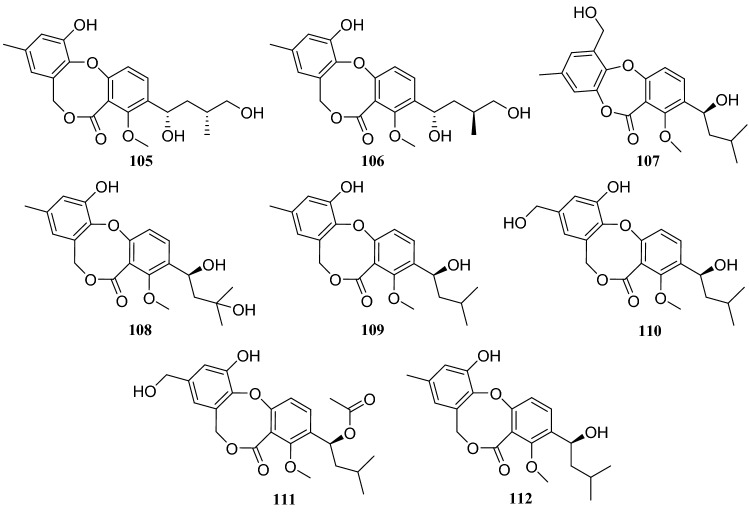
Structures of compounds **105**–**112**

**Fig. 13:**
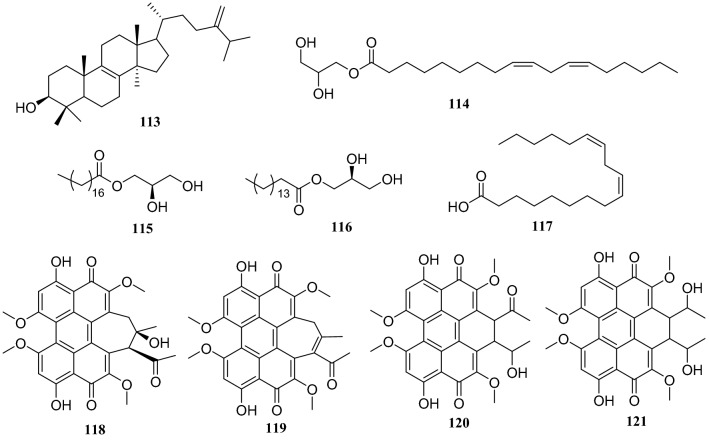
Structures of compounds **113**–**121**

The systematic names of the two lanostane-type triterpenoids are 3-carboxyl-4,12*β*,28-trihydroxyl-3,4-seco-5*α*-27-ketone-lanostane (**139**), and 3-carboxymethyl-4,12*β*,28-trihydroxyl-3,4-seco-5*α*-27-ketone-lanostane (**140**). Four sterol derivatives were identified as compounds **31**, **39,** 3*β*,5,8-trihydroxy-D-homo-ergosterol (**141**) and (20*S*,22*E*,24*R*)-ergosta-7,22-dien-3*β*,5*α*,6*β*-triol (**142**). Furthermore, the evaluation of all compounds for antitumor and antibacterial activities showed that only compound **139** showed weak cytotoxicity, with an inhibition rate of 21.1% at 20 μg/mL, whereas others showed no apparent activity [41] (Figures 11, 12, 13).

## *Dysosma versipellis* (Hance) M. Cheng ex Yang

*Dysosma versipellis* is an herbaceous perennial species that grows in the understory of mixed evergreen and deciduous forests in China. As an important endangered medicinal plant species, *D. versipellis* is restricted to eastern and southern China [42]. A strain of the *Penicillium sp*. was isolated and cultured from the fresh leaf of *D. versipellis*. Studies on the metabolites of the crude extract led to the isolation of the following twelve compounds: seven meroterpenoids, 11*β*-acetoxyisoaustinone (**143**), austin (**144**), austinolide (**145**), dehydroaustinol (**146**), dehydroaustin (**147**), chrodrimanin A (**148**) and chrodrimin B (**149**), a butyrolactone, isoberkedienolactone (**150**), and four other types of compounds, *O*-methylmellein (**151**), 3-(propan-2-ylidene)-pyrrolidine-2,5-dione (**152**), (*E*)-3-[2,5-dioxo-3-(propan-2-ylidene)-pyrrolidin-1-yl]acrylic acid (**153**) and *N*-(4-hydroxy-2-methoxyphenyl)-acetamide (**154**). All the compounds were evaluated for cytotoxicity in vitro using the MTT method but they only showed weak cytotoxicity [43].

**Fig. 14:**
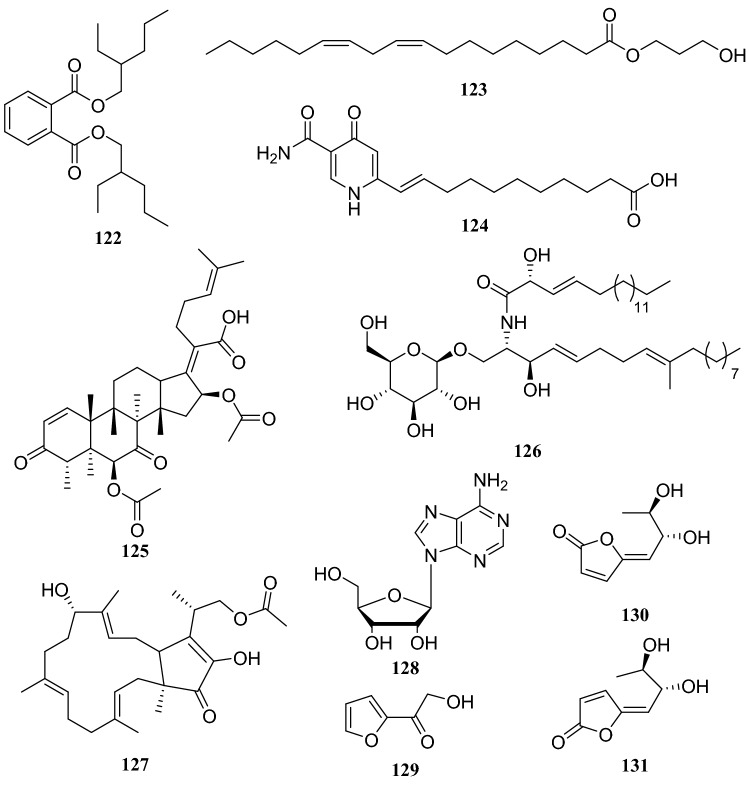
Structures of compounds **122**–**131**

Sixteen compounds isolated from an endophytic fungus *Paecilomyces Bainer* isolated from the roots of *D. versipellis* were as follows: nine sterols, **15**, **39**, cholesterol, 5,8-epidioxy-5*α*,8*α*-ergosta-6,9,22*E*-tien-3*β-*ol (**155**), ergosta-4,6,8(14),22-tetraene-3-one (**156**), ganodemaside B (**157**), 5*α*,6*α*-epoxy-3*β*-hydroxy-(22*E*)-ergosta-8(14), 22-dien-7-one (**158**), stigmasterol (**159**) and (*Z*)-stigmasta-5,24(28)-dien-3*β*-ol (**160**), two fatty acids, hexadecenoic acid (**161**) and oleic acid (**162**), two glycerides glycerol monooleate (**163**) and one nucleotide (**128**). In MTT assay, these compounds showed some cytotoxicity, and compound **157** showed strong cytotoxicity [44] (Fig. 14).

## *Celastrus anglatus* Maxim

**Fig. 15:**
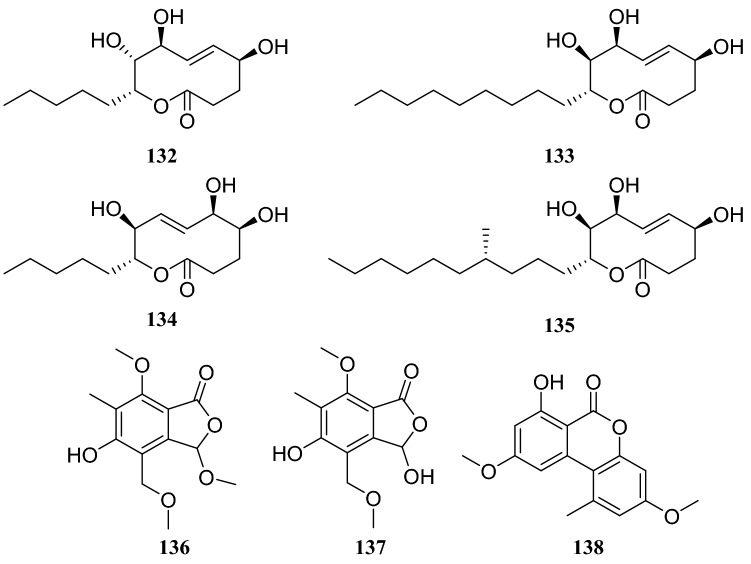
Structures of compounds **132**–**138**

*Celastrus anglatus*, which is heavily distributed in the mountains of southwest China, has been exploited as a natural insecticide resource and is a popular ingredient in folk medicine because of its active ingredients [45]. The following three compounds were isolated from the endophytic fungus *Oospora Wallr*, which was isolated from *C. anglatus*: **31**, cytochalasin D (**164**), and ducitol (**165**). All compounds were evaluated for their inhibitory activity against plant pathogens on spore germination at a concentration of 100 μg/mL. The inhibitory activity of compound **164** was strong, with a half-maximal effective concentration (EC_50_) of 35.01 μg/mL against *Alternaria longipes* [46]. Bioassay-guided fractionation led to the isolation of five antibacterial compounds from the fermentation broth of unknown endophytic fungus isolated from *C. anglatus*. These compounds were identified as 3′-chlorotrypacidin (**166**), asterric acid (**167**), methylasterrate (**168**), methyl-4′,6′-dichloroasterrate (**169**), and methyl-4′-chloroasterrate (**170**). The inhibition rates of compounds **165** and **166** (at 500 μM) against *Curvularia lunata* were 100% and 67.6%, respectively, and they showed strong inhibitory activity against *Bacillus subtilis* [47]. An endophytic fungus isolated from the phloem of *C. anglatus* was identified as *Fusarium proliferatum* and three cyclopeptides isolated from this strain were named enniatin A1 (**171**), enniatin B1 (**172**) and enniatin B (**173**) [48] (Fig. 15)

## *Camptotheca acuminata* Decne

**Fig. 16:**
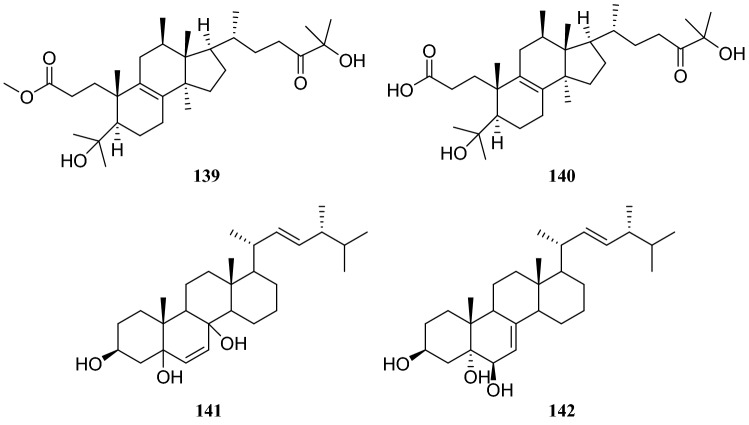
Structures of compounds **139**–**142**

*Camptotheca acuminata* is a tree species indigenous to southern China, which is referred to as “xi shu” and is of particular interest because of its importance of secondary metabolite, camptothecin and its analogs. Camptothecin (**174**) is known for remarkable inhibitory activity against tumor cells and the human immunodeficiency virus (HIV) [49]. Endophytic fungi can produce the same or similar metabolites as the host plant and, therefore, **174** and its derivatives, 9-methoxycampothecin (**175**) and 10-hydroxycampothecin (**176**), have been obtained from several endophytic fungi isolated from *C. acuminata.* (Table 1) [50–55] (Fig. 16)

**Table 1:** Camptothecin and its derivatives from endophytic fungi

Endophytic fungus	Compounds
*Fusarium solani*	**174**, **175**, and **176**
*Xylaria* sp.	**176**
*Mycelia sterlia*	**176**
*Botryosphaeria berengeriana*	**176**
*Diaporthe phaseolorum*	**176**
*Penicillium* sp.	**174**
*Alternaria* sp.	**174**
*Phomopsis* sp.	**174**
*Fusarium* sp.	**174**
*Colletotrichum* sp.	**176**

**Fig. 17:**
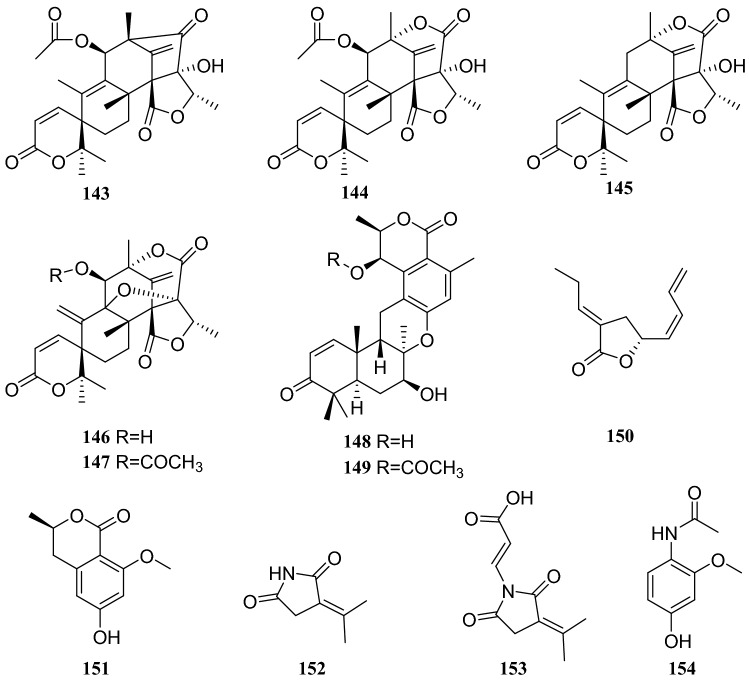
Structures of compounds **143**–**154**

Chemical investigation of the secondary metabolites of a fermented endophytic fungus of the *Aspergillus * sp. isolated several complex alkaloids. These compounds were identified as pseurotin A (**177**), FD-838 (**178**), fumitremorgin C (**179**), cyclotryprostatin B (**180**), 12,13-dihydroxyfumitremorgin C (**181**), fuminquinazoline C (**182**), fumiquinazoline J (**183**), spirotryprostatin A (**184**) and tryprostatin B (**185**). This fungus was isolated from the inner bark of *C. acuminata* [56] (Fig. 17).

Tan et al. [57] reported the isolation of five 10-membered macrolides and an unsaturated fatty acid and its methyl ester from the fermentation products of the endophytic fungal strain *Phomopsis* sp. isolated from *C. acuminata*. The seven compounds were identified as 8-*O*-acetylmultiplolide A (**186**), multiplolide A (**187**), 8-*O*-acetyl-5,6-dihydro-5,6-epoxymultiplolide A (**188**), 5,6-dihydro-5,6-epoxymultiplolide A (**189**), 3,4-deoxy-didehydromultiplolide A (**190**), (4*E*)-6,7,9-trihydroxydec-4-enoic acid (**191**), and (4*E*)-6,7,9-trihydroxydec-4-enoate (**192**). Furthermore, five 10-membered macrolides, **186**–**190**, exhibited no obvious antifungal activity against *C. albicans* at 200 μg/mL. The cytotoxic activities of compounds **186–189** against human-tumor *Raji* cells were tested using the MTT assay, but none exhibited cytotoxicity. Only compound **186** exhibited significant inhibitory activity against AChE, with an IC_50_ of 1.19 μg/mL.

**Fig. 18:**
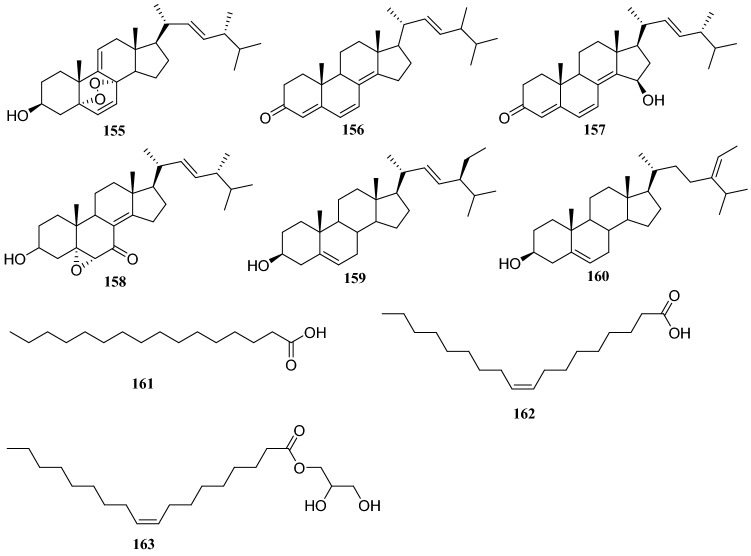
Structures of compounds **155**–**162**

Studies on the secondary metabolites from an endophytic fungus of the *Diaporthe * sp. isolated from surface-sterilized twig tissues of *C.acuminata*, led to the discovery of four new polyketides, *rel-*(2*R*,3*S*,4*R*,5*R*)-4-ethyltetrahydro-3-methyl-5-propylfuran-2,3-diol (**193**), methyl 5-[(1*R*)1-hydroxyethyl]-g-oxofuran-2-butanoate (**194**), butyl 5-[(1*R*)-1-hydroxyethyl]-γ-oxofuran-2-butanoate (**195**), 5-[(1*R*)-1-hydroxyethyl]-γ-oxofuran-2-butanoic acid (**196**), 3,4-dihydro-5′-[(1*R*)-1-hydroxyethyl][2,2′-bifuran]-5(2*H*)-one (**197**), phomopsolide B (**198**), and (2*S*,3*S*)-3,6-dihydro-6-oxo-2-{(1*E*)-2-[(4*S*,5*S*)-2,2,5-trimethyl-1,3-dioxolan-4-yl]ethenyl}-2*H*-pyran-3-yl (2*E*)-2-methylbut-2-enoate (**199**). In biostudies, compound **198** showed strong cytotoxicity against human-tumor HeLa cells, with an IC_50_ of 0.019 μm/mL. Compounds **196**, **198** and **199** were tested for their antibacterial and antifungal activities and at a concentration of 50 μg/disk, compound **198** showed an inhibitory zone with diameter of 1.4 cm against *Shigella dysenteriae* [58] (Fig. 18).

**Fig. 19:**
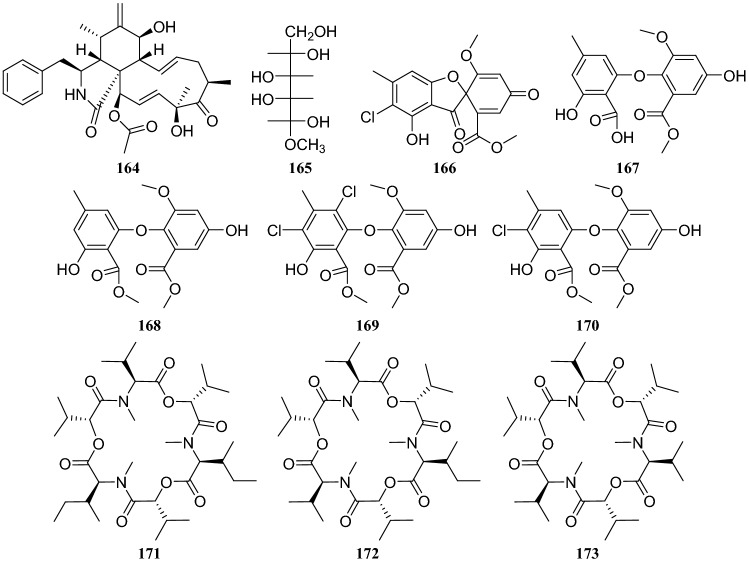
Structures of compounds **164**–**173**

Research on the secondary metabolites from an endophytic fungal strain of the *Phomopsis *sp., which was also isolated from the surface-sterilized twig tissues of *C. acuminata*, led to the isolation of 15 compounds: eleven naphthalene-type fungal polyketides, oblongolides B, C, N, O, P, Q, R, S, T, U, V (**200–210**); two new linear furanopolyketides named 5-{5-[(*R*)-1-hydroxyethyl]furan-2-yl}dihydrofuran-2(3*H*)-one (**211**) and 5-{5-[(*R*)-1-methoxyethyl]furan-2-yl}dihydrofuran2(3*H*)-one (**212**); one meroterpene named dihydroxysabinae (**213**); and one sesterterpene terpestacin (**214**). Furthermore, all compounds except **206**, **208**, and **210** were tested for their antimicrobial activities, but none showed a substantial effect [59] (Fig. 19).

**Fig. 20:**
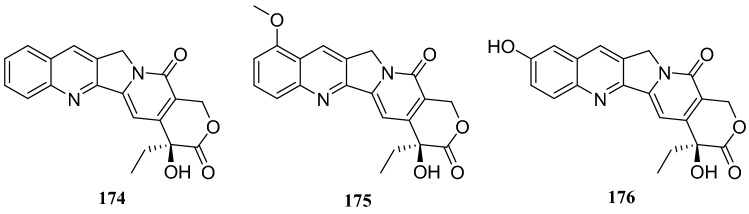
Structures of compounds **174**–**176**

From an endophytic fungus of the *Phomopsis* sp., isolated from *C. acuminata*, the following 12 compounds were isolated: oblongolide compounds **200**, **201**, **203**, **204**, **209**, oblongolides C1 (**215**), D (**216**), P1 (**217**) and X1 (**218**), a phomodiol 6-hydroxyphomodiol (**219**), (3*R*,4*R*,5*S*,6*R*)-6-hydroxy-5-methylramulosin (**220**), and (3*R*)-5-methylmellein (**221**). Some oblongolides previously obtained from this endophytic fungus were isolated from another fungus of the *Phomopsis* sp. All the compounds were evaluated for their cytotoxicities. Compounds **215**, **217**, **218**, and **219** exhibited modest selective activities against the HepG2 cancer cell lines, and compound **201** showed minor selective activity against A549 cells[60]. Compounds **85**, **86**, **204**, **210**, **211**, **212**, **216**, and oblongolide H (**222**) were isolated from a different endophytic fungus of the *Diaporthe* sp. [61] (Fig. 20)

**Fig. 21:**
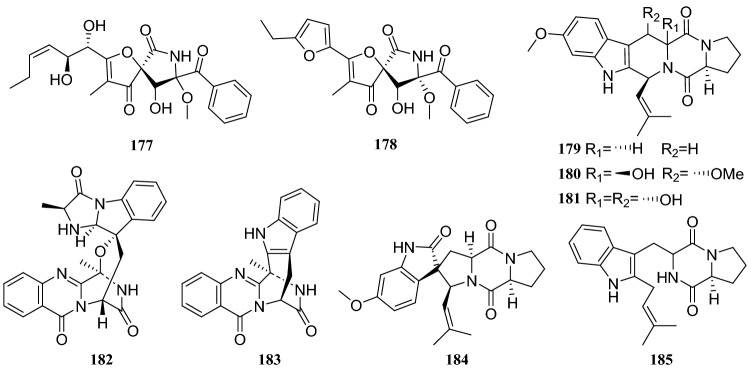
Structures of compounds **177**–**185**

Primary chemical profiling of *C. acuminata*-derived endophytic fungus *Penicillium polonicum* isolated nine compounds. Based on the nuclear magnetic resonance (NMR) and MS data, they were identified as polonicin A (**223**), polonicin B (**224**), 1233A (**225**), fusarubin (**226**), 3-methy ether-fusarubin (**227**), 5,8-dihydroxy-3-methoxy-7-methyl-6-(2-oxopropyl-1,4-naphthoquinone (**228**), anhydrofusarubin (**229**), 2-isopropanol-3-methyl-7-methoxy-naphthazarin (**230**) and 5-hydroxydihydrofusarubin D (**231**). All compounds were evaluated against the HepG2 hepatocellular carcinoma cell lines and compounds **228**–**231** showed cytotoxicity against the HepG2 cells. Additionally, all compounds were evaluated for their antidiabetic activity against L6 cells at a concentration of 30 μg/mL and compounds **223**, **224**, and **225** increased the rate of glucose uptake by 1.8, 1.5 and 1.25 times, respectively. Moreover, incubation of L6 cells with compound **223** stably increased the fluorescence intensity on the membranes by 2.1fold [62] (Fig. 21).

**Fig. 22:**
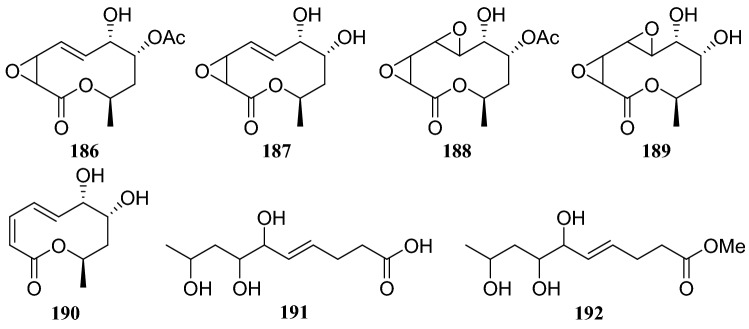
Structures of compounds **186**–**192**

Two lactone derivatives were isolated from an endophytic fungus of the *Diaporthe* sp. cultivated on *C. acuminata*, and identified as 5-([*E*]-1,4,5-trihydroxyhex-2-enyl)furan-2(5*H*)-one (**232**) and (5*Z*)-5-(2,3,4,5-tetrahydroxyhexyidene)furan-2(5*H*)-one (**233**). An MTT assay of compound **232** showed its antitumor activity against human cervical carcinoma cells Hela, and compound **233** exhibited strong inhibitory effects against MCF-7 breast cancer cells, human SH-SY5Y neuroblastoma and Lewis 3LL lung carcinoma cells [63] (Fig. 22).

## *Paris polyphylla* Smith

**Fig. 23:**
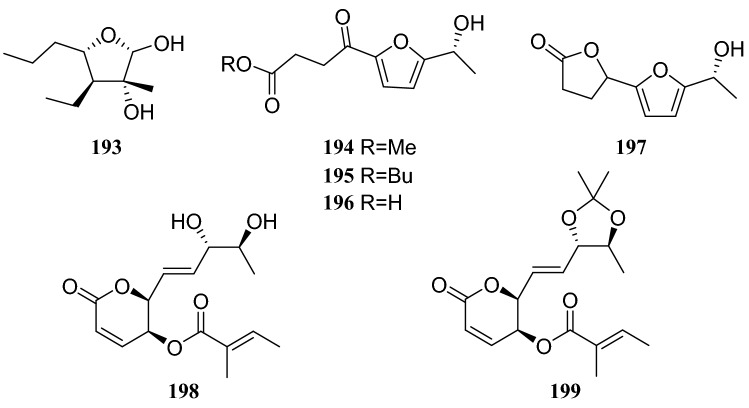
Structures of compounds **193**–**199**

*Paris polyphylla* is a perennial medicinal plant that has been used to treat traumatic injuries such as bleeding and snake bites as well as conditions such as carbuncles, furuncles, scrofula, and chronic bronchitis [64]. Bioassay-guided fractionation of the EtOAc extract of an endophytic fungus of the *Penicillium* sp. led to the isolation of eight polyketides: **90**, 1,3,4-trimethoxyl-6-methyl-9,10-anthraquinone (**234**), bostrycin (**235**), isorhodoptilometrin (**236**), physcioin (**237**), emodin (**238**), aloesol (**239**), and coniochaetone B (**240**). This endophytic fungus was isolated from the leaves of the *Paris polyphylla.* Furthermore, eight compounds were evaluated for antitumor activity against the HepG2 cell line, and only compounds **234**–**236** showed inhibitory activity, with IC_50_ of 15.6, 6.5, and 13.2 μg/mL, respectively [65]. Five sterols with unusual bicyclo [4.4.1] skeletons isolated from the same strain of a *Penicillium* sp. were identified as 22-acetyisocyclocitrinol A (**241**), neocylcocitrinols A (**242**), B (**243**), C (**244**), and D (**245**) [66] (Fig. 23).

**Fig. 24:**
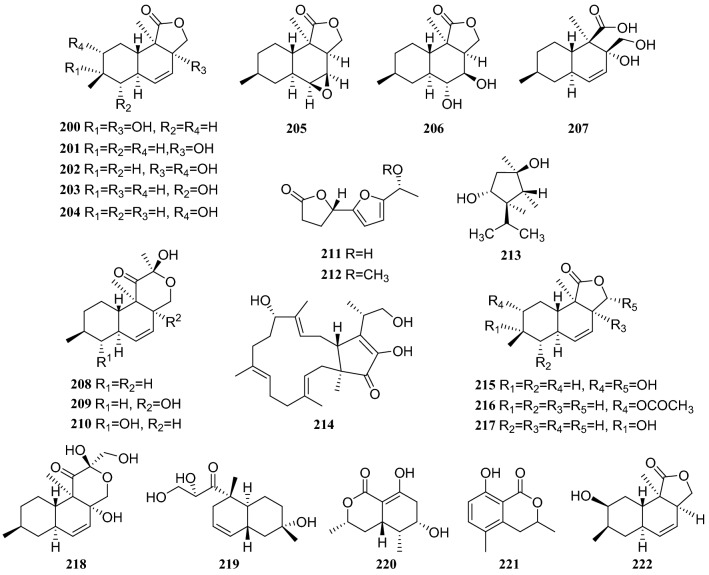
Structures of compounds **200**–**222**

Eight compounds isolated from another strain of a *Penicillium* sp. isolated from *Paris polyphylla*, were identified as **109**, citrinin H1 (**246**), dehydroisopenicillide (**247**), 7-en-nonadecanoic acid monoglyceride (**248**), 7,9-dien-nonadecanoic acid monoglyceride (**249**), silbaticol (**250**), 5-hydroxy-2-pyridinemethanol (**251**), and 2,4,6-octatrienoic acid (**252**). In an MTT assay evaluating eight compounds in HepG2 cell lines, **109**, **246**, **247**, and **251** showed inhibitory activity with IC_50_ values of 8.5, 12.5, 15.0, and 18.2 μg/mL, respectively [67] (Fig. 24)

## Miscellaneous

**Fig. 25:**
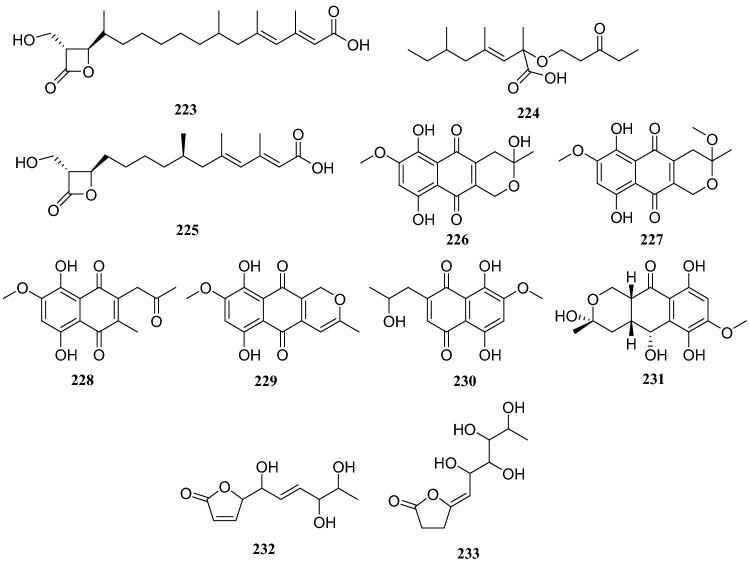
Structures of compounds **223**–**233**

Two known metabolites isolated from the endophytic fungus *Trichoderma ovalisporum* obtained from *Caesalpinia decapetal* were identified as **163** and (*Z*)-9-heptadecenoic (**253**) [68]. Five known alkaloids were isolated from the endophytic fungus *Fusarium oxysporum* obtained from the roots of *Iris tectorum*. The compounds were identified as beauvericin (**254**), 4-oxopentanoic acid (**255**), *N*-(4-oxopentyl)-acetamide (**256**), 5-butyl-2-pyridinecarboxylic acid (**257**) and 5-butylene-2-pyridinecarboxylic acid (**258**). In biostudies, only beauvericin showed strong antibacterial activity against *S.aureus* and *E.coli.* All compounds were evaluated for their cytotoxic activity against HepG2, HepG3 and LO2 cells using an MTT assay. Among evaluated compounds, beauvericin, 5-butyl-2-pyridinecarboxylic acid and 5-butylene-2-pyridinecarboxylic acid exhibited weak antitumor activity, with IC_50_ of 65.3–120.5 μg/mL, as well as weak cytotoxic activity against LO2 cells [69]. Bioassay-guided fractionation of an EtOAc extract of the endophytic fungus *Bionectria ochroleuca* led to the isolation of a known compound glutaric acid methyl ester (**259**), along with seven unattuned compounds. This endophytic fungus was isolated from *Vitex negundo* [70]. Several endophytic fungi, including *Fusarium oxysporum*, *Trichoderma hamatum*, and *Fusarium* sp., isolated from the *Paris polyphylla* Sm. var chinensis (Franch.) can produce diosgenin (**260**) [71] (Fig. 25).

## Conclusion

**Fig. 26:**
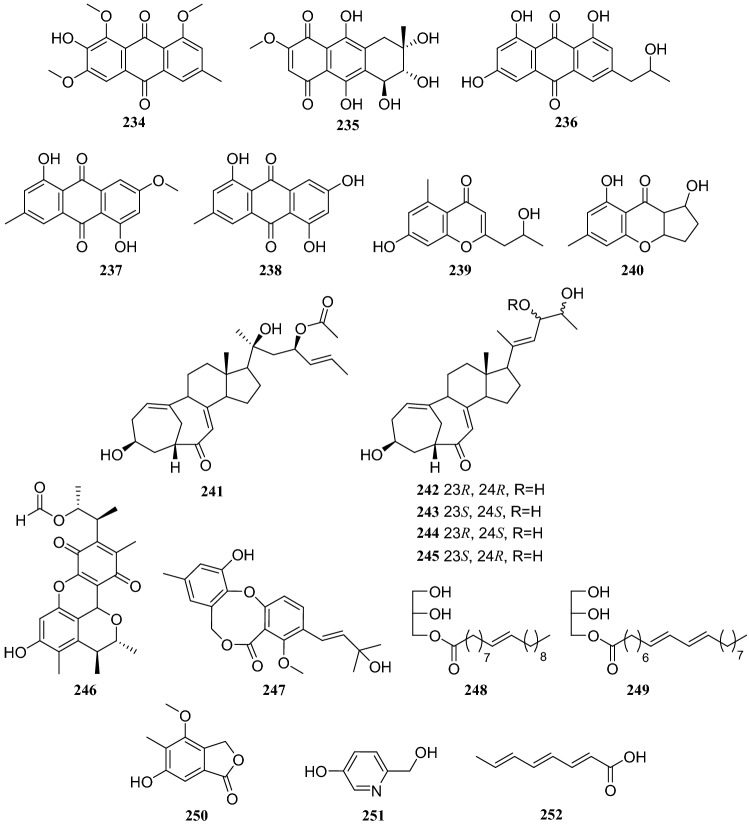
Structure of compounds **234**–**252**

It is important to point out that endophytic fungi produce highly diverse secondary metabolites and, therefore, could be used to as sources to discover novel natural products with important bioactivities. Considering the vulnerability and limitation of productivity of plants, endophytic fungi are a potential renewable and inexhaustible source of novel drugs and agrochemicals (Fig. 26).

**Fig. 27:**
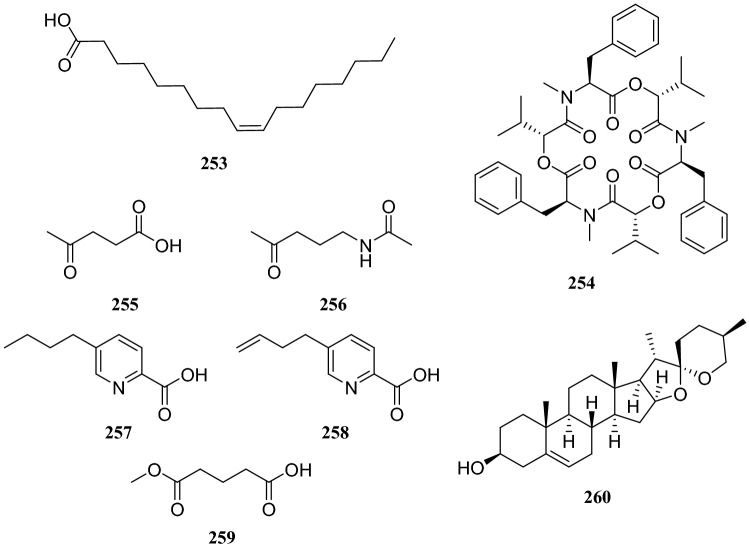
Structures of compounds **253**–**260**

Interactions between endophytic fungi and host plants are established through complex chemical and biological networks. Endophytic fungi inhibiting plants can colonize their internal tissues without causing disease symptoms. The plant hosts in mutualistic symbioses provide favorable conditions for endophyte development. The microorganisms can produce the same compounds found in the medicinal plants, probably because an exchange of genetic material occurs between the endophyte and plant. Studying and understanding these interactions is essential to achieving the sustainable production of natural products with significant bioactivities from endophytic fungi [72] (Fig. 27).

In Hubei province, over 2000 natural medicinal plants are used in Tujia medicine [73]. Because their overdevelopment and overuse, many medicinal plants are becoming scare, and some are facing extinction. Thus, the use of medicinal plants for the isolation of endophytic fungi is one conservation options. Moreover, endophytic fungi may significantly reduce the use of agrochemicals (fertilizers, fungicides, insecticides, and herbicides) in the cultivation of medicinal plants. The loss of endophytic microbes from medicinal plants during cultivation could be mediated by the transfer of endophytes from wild relatives of medicinal plants to cultivated species. Furthermore, endophytes from medicinal plants used in Tujia medicine have been poorly investigated and should not be neglected because of their natural origin. Finally, the study of endophytic fungi as a renewable source is in its infancy, and should be further explored in future research.
